# The Importance of Craniofacial Sutures in Biomechanical Finite Element Models of the Domestic Pig

**DOI:** 10.1371/journal.pone.0031769

**Published:** 2012-02-21

**Authors:** Jen A. Bright

**Affiliations:** Department of Earth Sciences, University of Bristol, Bristol, United Kingdom; Raymond M. Alf Museum of Paleontology, United States of America

## Abstract

Craniofacial sutures are a ubiquitous feature of the vertebrate skull. Previous experimental work has shown that bone strain magnitudes and orientations often vary when moving from one bone to another, across a craniofacial suture. This has led to the hypothesis that craniofacial sutures act to modify the strain environment of the skull, possibly as a mode of dissipating high stresses generated during feeding or impact. This study tests the hypothesis that the introduction of craniofacial sutures into finite element (FE) models of a modern domestic pig skull would improve model accuracy compared to a model without sutures. This allowed the mechanical effects of sutures to be assessed in isolation from other confounding variables. These models were also validated against strain gauge data collected from the same specimen *ex vivo*. The experimental strain data showed notable strain differences between adjacent bones, but this effect was generally not observed in either model. It was found that the inclusion of sutures in finite element models affected strain magnitudes, ratios, orientations and contour patterns, yet contrary to expectations, this did not improve the fit of the model to the experimental data, but resulted in a model that was less accurate. It is demonstrated that the presence or absence of sutures alone is not responsible for the inaccuracies in model strain, and is suggested that variations in local bone material properties, which were not accounted for by the FE models, could instead be responsible for the pattern of results.

## Introduction

The skulls of vertebrates are composed of many individual bones joined together at craniofacial sutures, synarthrotic intersections between the bones that are bridged by collagen fibres. These sutures are of particular importance during ontogeny, because bone is deposited at the sutural junctions, permitting growth. During growth, sutures tend to become more ossified, or “fused”, with premature fusion associated with growth disruption and craniofacial malformation [1]. In many reptiles and birds, sutures are also an important mechanical feature of the skull, acting as kinetic joints that experience significant movement during feeding and other behaviours [2]. Thus, in these animals, it is common for some sutures to remain unfused at maturity. In mammals such kinesis at the sutures is not observed, and sutures often fuse once the bones have reached their adult size. However, some mammalian sutures remain patent well into adulthood [3].

The fact that some mammalian sutures remain patent, apparently introducing a zone of weakness into the skull, has led to the hypothesis that sutures may have a functional role [4]–[6]. In support of this, sutural morphology can often be used to predict the dominant type of strain (compressive or tensile) that a suture experiences, with interdigitated sutures being indicative of a compressive strain regime, and simple, butt-ended or bevelled sutures indicating tension [3], [4], [7], [8]. The arrangement of collagen fibres in these two morphotypes is consistent with this hypothesis, because the fibres are well orientated to bear such strains [7], [9]. More complex suture interdigitation has also been associated with increased loading in the skull, either from the masticatory muscles [10], a diet comprising resistant foods [11], or possibly from a strong genetic signal [12].

Extensive experimental work using strain gauges has measured deformation in the skulls of several mammalian taxa both *in vivo* and *ex vivo*. In multiple *in vivo* experiments on miniature pigs, Herring and colleagues have found that strain magnitudes and orientations on adjacent bones in the skull are often different, and that strain in sutures is higher than that in bones [4], [7], [13]–[16]. These authors suggest that patent sutures may therefore act as strain modifiers, possibly protecting the more delicate bones in the face from high stresses developed during feeding. Similar results, showing that adjacent bones can have notably different strain magnitudes and orientations, have been obtained in sheep [17] and macaques [18]. Impact loading and bending tests performed *ex vivo* on the bones and sutures of goats [5], [6] show that sutures absorb more energy than bones upon impact, and the ability of sutures to do so is positively correlated with the degree of interdigitation. It has therefore been suggested that sutures may play a dynamic role, acting as shock absorbers during forceful movements such as head-butting [5], [6], [19].

If craniofacial sutures are performing a functional role, then their inclusion in biomechanical models of the skull may be crucial. Finite Element Analysis (FEA) is an engineering technique that allows the quantification of various performance metrics in complex shapes, by discretising their geometry into an interconnected mesh of small, geometrically simple bricks (elements). By treating each element as a term in a simultaneous equation, and solving for stress and strain, an approximation of the stresses and strains in the continuum structure can be reached [20], [21]. Because material properties, loading regimes and geometries are easily manipulated within the modelling environment, FEA is a widely used technique to investigate the associations between skeletal function and form in both living and extinct taxa [21], [22]. Therefore, FE models potentially offer a powerful method for testing the biomechanical significance of a number of skeletal features, including craniofacial sutures. If sutures are demonstrated to play a significant role in skull function, yet are not appropriately considered in FE models, then there is the potential for such models to give results that may be inaccurate or misleading [18], [21], [23], [24].

So far, FEA investigating the role of craniofacial sutures has been ambiguous. Kupczik et al. [25] validated a FE-model of a macaque skull including the zygomatic suture by comparing their FE model with *ex vivo* strain gauge data. Sutures were modelled as fused, open (as a break in the mesh), or regions of flexible 3D elements with varied material properties. Finite element strain results in the zygomatic arch gave a better approximation of *ex vivo* strains when flexible 3D elements were modelled with sutural properties measured from their specimen by nanoindentation. But, this came at the expense of higher strain elsewhere in the face. Conversely, in a model incorporating four facial sutures bilaterally, Wang et al. [26] found that sutures made virtually no difference to the patterns of strain in a macaque skull when compared with a solid model (although the suture models were more flexible and experienced higher deformations). This study therefore suggested that whether macaque sutures are modelled as open or fused probably has little effect on the reporting of stress or strain results of a finite element analysis. Indeed, other FE models of macaques have demonstrated reasonable correlation with *in vivo* experimental strain data [27]–[29], reporting strain ratios and orientations within the experimentally measured range, despite the fact that sutures were omitted from these models. Moazen et al. [23] modelled a reptilian (*Uromastyx*) skull with multiple sutures, again with the sutures modelled as flexible regions of 3D elements. Local perturbations in strain were observed when compared with a solid FE-model, but overall strain was not substantially reduced. They suggested that sutures may act to relieve local strains in a number of ways depending on the type of loading encountered, by redistributing strains so that they are equalised throughout the whole skull. Again, this study seems to indicate that sutures act to reroute stresses, causing nearby bones to experience either a decrease or increase in strain. Reed et al. [24] also found that changing the stiffness of 3D elements representing sutures in models of the alligator mandible significantly affected the strain regime both in individual bones, and in the whole model. As reptiles and birds have more bones in their crania compared to mammals, and have a greater proportion of unfused sutures connecting these bones, it is possible that these differences in results are a function of the taxa that have been studied.

Bright and Gröning [30] validated a method for FE-modelling of sutures by studying the zygomatic arch of a domestic pig using digital speckle pattern interferometry (DSPI), and found that the most accurate means of capturing sutural mechanical behaviour was to model sutures as regions of flexible, 3D elements. Their experiment also indicated that high strain gradients are observed across the suture, but these are localised and do not affect bone beyond a few mm from the suture. The suture did however allow the two bones in the zygomatic arch to move independently of one another. It is therefore possible that the cumulative effect of multiple small displacements at the sutures may be sufficient to change the structural behaviour of the whole skull when compared to a model with no sutures. In an additional study [31], it was found that a solid FE-model of the pig skull did not always correctly report the magnitudes of strain, although it could be shown that the model was experiencing a similar loading condition to the *ex vivo* experiment. However, a general fit of experimental strain ratio and orientation to FE-results was achieved. It was noticed that areas that failed to accurately report strain were often located near sutures. Other finite element workers have also suggested that neglecting to model craniofacial or mandibular sutures may have been responsible for local lack of fit between their experimental and modelled data [27], [32].

The aim of this study was to compare finite element models of a modern domestic pig skull that either did or did not incorporate craniofacial sutures. This data was also compared to experimental strain data gathered *ex vivo* to assess how sutures influence model validity. Here, the hypothesis that introducing craniofacial sutures into the model will improve the fit of experimental to model data is tested by incorporating six prominent sutures into a model validated with *ex vivo* strain gauge data.

## Materials and Methods

Experimental strain data for comparison with the models was collected from a pig specimen *ex vivo*. The experimental set-up has been described previously in Bright and Rayfield [31], and a summary is given below. Although a considerable body of research on bone and suture strain in the pig skull has been collected *in vivo* by Herring and colleagues [4], [7], [8], [13]–[16], there are several reasons why it was not appropriate to validate the models against Herring et al.'s data. Briefly, the objective of this study was to perform a specimen-specific validation, so that the effects of intraspecific differences in morphology and bite kinematics did not confound the results arising from inaccuracies in model input parameters. This raises several problems with using *in vivo* data: Firstly, it was desired that the experimental loading was precisely known, and that this load was repeatable in the FE-model. As such, a comparison with strain data collected *in vivo* from a number of individuals with unknown muscle force inputs and bite points was unsuitable for this purpose, and a simplified *ex vivo* loading condition was preferred. For these same reasons, the range of strain values that are generated for any given gauge site *in vivo* tend to show huge variability in their results, sometimes having a standard deviation greater than 50% of the mean value [14]. Conversely, the standard error of the *ex vivo* strain data utilised in this study was in the range of 5–10% of the mean value [31]. The greater precision of *ex vivo* experimental data, and the fact that the FE-model should be able to precisely replicate the boundary conditions of the experiment, makes an *ex vivo* dataset much more suitable for validation in this instance. Furthermore, the lack of precision in being able to place gauges in homologous locations to those of Herring et al. would have exaggerated these effects. Finally, Herring et al. have worked exclusively with Hanford strain laboratory-reared miniature pigs. The breed of pig in this investigation was a farm-reared Large White Breed, which is of a vastly different size, and different morphology, which again will exaggerate all of the issues raised above.

A load of 755 N was applied to the fresh pig skull specimen (Large White Breed, age approx. six months, defleshed skull dimensions 247×141×133 mm) at the masseter and temporalis attachment sites using a custom-built testing rig. The load was approximately equal on the left and right side of the face, but was asymmetrically divided between the masseter and temporalis muscles, because pigs recruit the ipsilateral masseter and contralateral temporalis together to close the jaw [33], even when biting bilaterally. This arrangement pulled the specimen down onto bilateral supports at the teeth and temporomandibular joints (TMJ). Strain was recorded on 16 planar rosette strain gauges (G1-16) glued to the skull (C2A-06-062LR-350, Vishay Micro-Measurements, Basingstoke UK). These are electronic components that experience changes in the length of wire as a change in resistivity, and can therefore be used to measure strain. The rosette configuration of the gauges allows the direction of maximum principal strain to be determined. During the experiment, it was noticed that G1 was drifting, and thus gave unstable results. Similarly, G6 gave unstable results, flipping between compressive and tensile strains [31]. These gauges are therefore excluded from further discussion.

**Figure 1:**
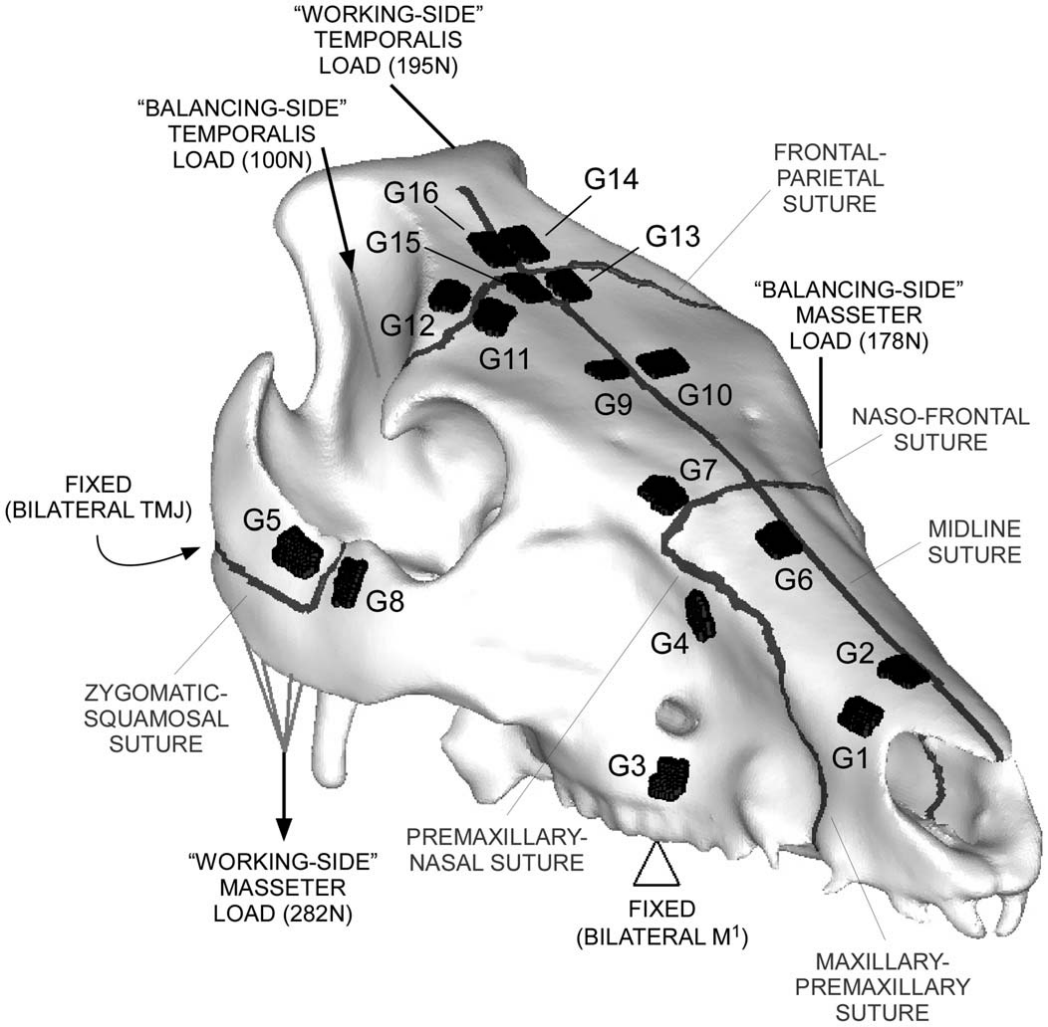
CT reconstruction of the pig skull for FE modelling. CT reconstruction of the pig skull, showing the locations and magnitudes of loading and constraints used in the FE model, as well as the positions of the strain gauges and sutures. TMJ = temporomandibular joint, M^1^ = 1^st^ molar tooth, G = gauge.

The same specimen was CT-scanned at the Royal Veterinary College on a Picker PQ5000 medical scanner (0.55 mm pixel size, 2 mm slice thickness, 120 kV, 200 mA), after which the slices were imported into *Amira 4.1* (Mercury Computer Systems Inc., USA), and a 3D surface of the bones was constructed and exported as a stereolithography (.stl) file. The .stl surface was imported into *HyperMesh 10.0* (Altair Engineering Inc., USA) for conversion into a finite element model. Loads were applied to the model via rigid body elements (RBE3 in *Abaqus*), and translational constraints applied to the teeth dorso-ventrally (Y) and in all directions at the TMJ (XYZ) to mimic the experimental set-up (Fig. 1). In the absence of material properties data for pig bone, the model was assigned the properties of human cranial cortical bone (E = 12.5 GPa, ν = 0.35 [34]), and was assumed to be isotropic and homogeneous (HOM model). It has already been shown that these assumptions result in a model that is too stiff (probably because it neglects to account for more flexible cancellous bone) but approximates the loading condition of the experiment well [31]. Because CT resolution was insufficient to accurately resolve the periodontal ligament (PDL), the teeth were modelled as being continuous with the bone, and assigned the same material properties. There is currently debate over whether or not the exclusion of the PDL results in models that are too stiff, and that deform differently [35]–[38], but neither issue should affect differences caused by the presence or absence of craniofacial sutures. Keeping these assumptions constant not only allows direct comparison with the results of the earlier study, but removes the potentially confounding effects of other variables in the model, allowing the effect of introducing sutures into FE models to be observed in isolation.

Convergence tests were performed on the solid model [39], and a mesh comprising 1,749,149 quadratic tetrahedral elements (TET10) was utilised. Six major sutures were inserted into the mesh: the right zygomatic-squamosal; midline; naso-frontal, frontal-parietal; and bilateral maxillary-premaxillary/maxillary-nasal sutures (Fig. 1). As these were poorly resolved by the CT scan (voxel sizes were 0.55×0.55×2 mm), sutures were introduced manually, using measurements from the specimen to place them, and detailed CT reconstructions from a different specimen [40] to appropriately replicate the internal geometry (such as overlap between bones). On the scale of this model, the degree of interdigitation of the sutures was not considered. Although it has been shown that interdigitation at sutures is related to the way in which they distribute strains [9], this has been shown to be of negligible concern at the scale of the whole skull [30].

Sutures were created (SUT model) by defining bands of 3D elements throughout the bone thickness that were at least three elements wide (1.5 mm). This has been demonstrated to be an accurate means of simulating strain and displacement patterns at sutural junctions [30], both for interdigitated and smooth sutures, and in compression and tension. Although this band is wider than sutures in reality, it is a necessary abstraction (which has been shown not to invalidate the model [30]), as otherwise strain gradients developed in the model at the suture-bone interface become artificially steepened. Sutures were assigned the properties of pig nasofrontal suture (E = 46 MPa, ν = 0.35; [16]). It has already been demonstrated that this value of sutural stiffness (46 MPa) is too flexible when compared with *ex vivo* experimental data from the zygomatic arch of this specimen [30]. This could be due to a number of factors, including differences in the species and age of pigs in the two studies, differing degrees of internal fusion in the sutures, and the fact that values from the nasofrontal suture may not be comparable with those from the zygomatic arch. An averaged set of material properties representing a “zone” of suture and bone material, such as was used by Farke [19], will result in a value that is intermediate between bone and suture, and may be a better representation of the actual material properties of this band. However, using this flexible value of suture allows the model to provide an indication of performance assuming that all the sutures are essentially patent, which again removes the confounding effects of partial fusion.

Because strain gauges are 2D components, they are only able to report strains in the plane of the gauge, which is a projection of the true, three-dimensional strain. To account for this, membrane elements were defined in the locations of the strain gauges (Fig. 1). These are two-dimensional elements defined with negligible thickness (0.01 mm) and material properties (E = 0.001 GPa, ν = 0.35), which move with the bone surface, thus projecting the 3D strains of the models into the 2D plane of the gauge. In previous isotropic models, results from 2D membranes and the underlying 3D elements have been comparable [31].

Once constructed, models were exported to *Abaqus 6.8.2* (Dassault Systèmes Simulia Corp., Providence RI, USA) for solving on a desktop PC (Windows 64-bit Vista Business, Intel Xeon ×5450 3.00 GHz CPU, 64 GB RAM). At each gauge location, values of principal strain magnitudes (ε_max_; ε_min_), ratios (ε_max_/|ε_min_|), orientations and maximum shear strain (γ_max_ = ε_max_−ε_min_) were taken from all elements that formed the 2D “gauge” membrane, and results from each gauge were averaged. These values were then compared with the experimental data set.

## Results

The strains obtained by the *ex vivo* experiment are reported in detail elsewhere [31], but are summarised here and in Table 1. Principal strain magnitudes range between +485 με and −606 με. Strains are highest in the zygomatic arch (G5, G8), the frontals (G7) and in the maxilla, dorsal to the toothrow (G3). Strains are lowest in the rostrum (G2) and the cranial vault (G9-16), although the two gauges on the right parietal (G12, G16) show elevated strains compared with other nearby gauges. Particularly striking is the result that, when moving from one bone to another (i.e. crossing a suture), large differences in principal and shear strain magnitude, strain ratio, and principal strain orientation are apparent over very small distances (compare in particular G5/G8 on the zygomatic arch; G11/G12 and G13-G16 on the frontal and parietal bones). Results extracted from the HOM and SUT models are presented in Table 2, and are discussed below.

**Table 1:** Experimental grand means over three repeated loadings, ±2 standard errors.

	G1	G2	G3	G4	G5	G6	G7	G8	G9	G10	G11	G12	G13	G14	G15	G16
	Dorsal premaxilla	Anterior nasal	Maxilla, dorsal to M^1^	Dorsal maxilla	Zygomatic	Posterior nasal	Right anterior frontal	Squamosal	Right midline, frontal	Left midline, frontal	Lateral right posterior frontal	Lateral right parietal frontal	Left posterior frontal	Left anterior parietal	Right posterior frontal	Right anterior parietal
Tension (ε_max_)	108±10	52±5	388±22	264±5	456±33	−8.4±15	485±13	68±5	81±3	40±3	60±3	151±5	17±1	21±3	23±2	120±17
Compression (ε_min_)	−6±6	−84±2	−375±33	−107±6	−288±17	−54±4	−196±12	−606±18	−122±9	−84±12	−132±6	−196±5	−176±11	−74±6	−57±7	−40±4
Orientation (angle from grid 1 to ε_max_ axis)	−78±3	−49±0.8	45±0.4	46±0.3	−85±0.6	24±9	28±0.8	−28±1	−24±1	−26±3	−53±2	−85±1	44±0.9	70±5	57±2	87±1
Strain Ratio (ε_max_/|ε_min_|)	14±5	0.62±0.05	1.04±0.04	2.48±0.16	1.58±0.02	−0.11±0.29	2.45±0.23	0.11±0.01	0.67±0.02	0.49±0.06	0.46±0.04	0.77±0.02	0.10±0.004	0.28±0.01	0.42±0.10	2.94±0.17
Shear (γ = ε_max_−ε_min_)	115±8	135±7	763±55	371±7	743±50	46±11	681±4	674±14	202±12	125±14	192±4	347±9	193±12	94±9	80±5	160±21

The values given in this table were also presented in Ref. 31 (reprinted with permission of John Wiley & Sons, Inc.).

**Table 2:** Finite element analysis results compared between the models with and without sutures.

	G1	G2	G3	G4	G5	G6	G7	G8	G9	G10	G11	G12	G13	G14	G15	G16
	Maximum Principal Strain (ε_max_)															
HOM	9.40	1.70	32.49	23.91	110.01	1.79	45.50	158.23	12.96	13.11	9.79	6.78	11.03	7.73	12.14	8.17
SUT	16.50	7.47	24.77	46.21	197.39	4.48	77.35	154.20	10.44	2.88	1.86	11.03	9.07	5.79	7.18	6.88
	Minimum Principal Strain (ε_min_)															
HOM	−4.98	−1.01	−36.51	−25.24	−36.32	−15.06	−44.73	−188.37	−25.64	−28.44	−18.71	−10.89	−25.11	−16.41	−20.91	−15.13
SUT	−8.67	−2.44	−33.55	−12.43	−43.69	−8.61	−48.61	−118.89	−17.70	−7.51	−23.88	−14.54	−33.08	−10.36	−27.59	−9.48
	Strain Ratio															
HOM	1.89	1.68	0.89	0.95	3.03	0.12	1.02	0.84	0.51	0.46	0.52	0.62	0.44	0.47	0.58	0.54
SUT	1.90	3.06	0.74	3.72	4.52	0.52	1.59	1.30	0.59	0.38	0.08	0.76	0.27	0.56	0.26	0.73
	Shear Strain (γ)															
HOM	14.38	2.72	68.99	49.15	146.33	16.85	90.24	346.60	38.61	41.56	28.50	17.66	36.14	24.14	33.05	23.30
SUT	25.17	9.90	58.32	58.65	241.08	13.09	125.96	273.10	28.14	10.39	25.66	25.56	42.15	16.15	34.77	16.35
	Maximum Principal Strain Orientation (°)															
HOM	−81.5	−47	45	45.5	−87.5	10.5	20	−67.5	−34	−37	−78.5	−84	47	45.5	47	46.5
SUT	−78	−57.5	50	75	−72	29	22	−72	−17	−33.5	−76	−72	44	36	51	67

**Figure 2:**
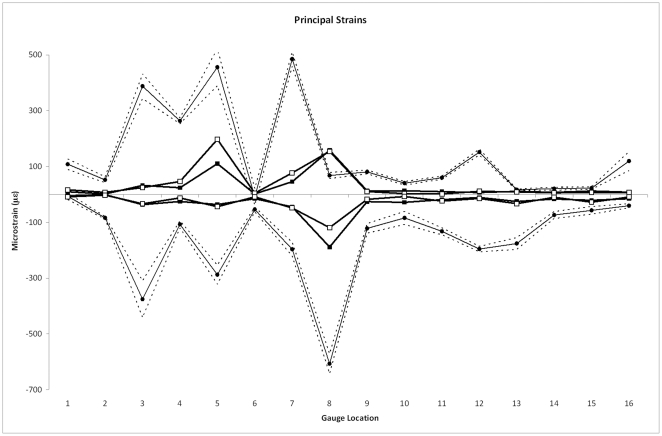
Comparisons of principal strains. Comparisons of principal strains in models without (black squares) and with (white squares) sutures, with experimental data (black circles). Dashed lines show 2 standard errors of the experimental mean. Gauges 1 and 6 gave unstable experimental results and should be disregarded.

Figure 2 compares the magnitudes of strains from the HOM and SUT models with the *ex vivo* experimental data. Firstly, the FE models are too stiff by approximately an order of magnitude. This was recognised by Bright and Rayfield [31], and shown to be largely the result of the omission of cancellous bone from the models, which lowers the overall stiffness (thus increasing strains) without affecting strain patterns.

Notably, it can be seen in Fig. 2 that there seems to be little difference between the HOM and SUT models in most locations, and that adding sutures does not induce strains that are more similar to the experimental results. Differences between the models can be seen at G4 (dorsal maxilla), G5 (temporal bone, zygomatic arch) and G7 (right anterior frontal), where ε_max_ is higher in the SUT model. Lower ε_min_ is seen in the SUT model at G4 (dorsal maxilla), G8 (zygomatic bone, zygomatic arch), and G10 (left midline, frontal).

**Figure 3:**
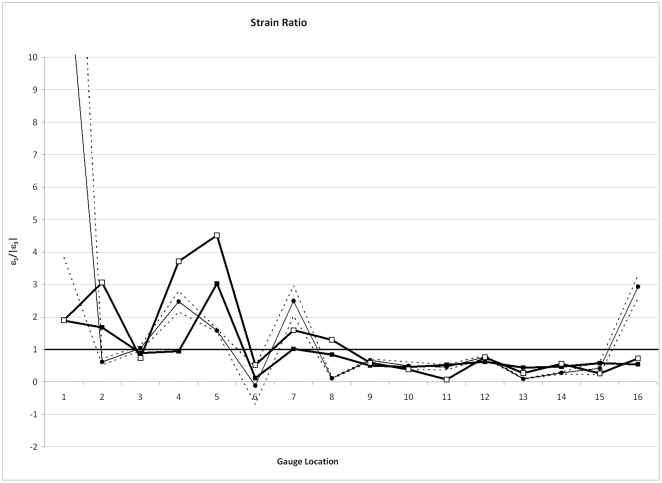
Comparisons of strain ratio. Comparisons of strain ratio (ε_max_/|ε_min_|) in models without (black squares) and with (white squares) sutures, with experimental data (black circles). Dashed lines show 2 standard errors of the experimental mean. Gauges 1 and 6 gave unstable experimental results and should be disregarded.

Because of the stiffness differences between the models and the experimental specimen, it is useful to observe strain ratio, as this removes the effect of strain magnitude (Fig. 3). Values >1 indicate tension is greater than compression and values <1 indicate that compression is greater than tension. Here, more differences between the models begin to show. Gauge 2 (anterior nasal) and G4-8 show increases in strain ratio in the SUT model, which, in the case of G4 (dorsal maxilla) changes the strain regime to one of overall tension. Gauge 11 has a lower value of strain ratio in the SUT model than the HOM model. In some cases (G4, G7), adding sutures brings the model closer to the experimental results, but in others (G2, G5, G8, G11) the model moves further from the experiment.

**Figure 4:**
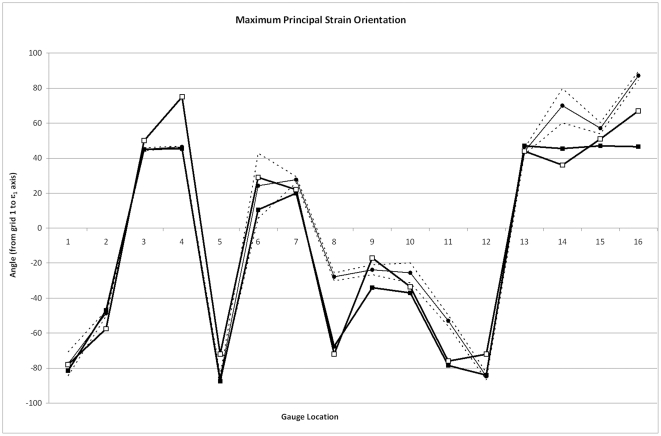
Comparisons of strain orientation. Comparisons of strain orientation in models without (black squares) and with (white squares) sutures, with experimental data (black circles). Dashed lines show 2 standard errors of the experimental mean. Gauges 1 and 6 gave unstable experimental results and should be disregarded.

Strain orientations between the experimental data set and the HOM model are remarkably consistent (Fig. 4, and see also [31]), indicating that this model represented the loading regime of the experiment well. The introduction of sutures has only a negligible effect (≤5° difference) in half of the gauge locations, but affects strain orientations by >5° in the other half (Table 2). For G6 (posterior nasal), G9 (right midline, frontal) and G16 (right anterior parietal), this represents an improvement, bringing the model closer to the experimental results, but in the other locations results are further from the experiment.

**Figure 5:**
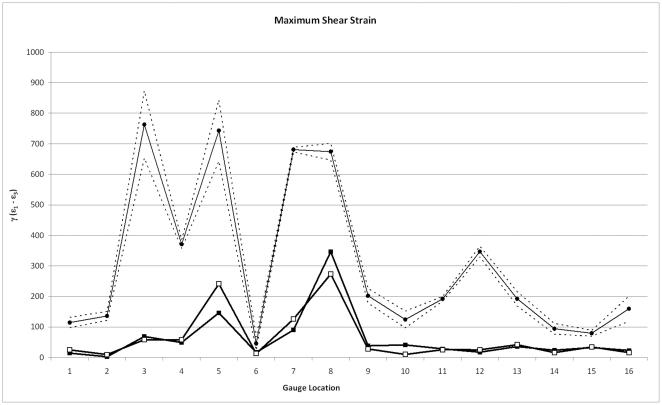
Comparisons of shear strain. Comparisons of shear strain (γ_max_ = ε_max_−ε_min_) in models without (black squares) and with (white squares) sutures, with experimental data (black circles). Dashed lines show 2 standard errors of the experimental mean. Gauges 1 and 6 gave unstable experimental results and should be disregarded.

As with the principal strains, the magnitudes of shear strain (γ_max_) are too low in comparison with the *ex vivo* results, but the models replicate two peaks of the experiment in the zygomatic arch, at G5 and G8, although not elsewhere at G7, G12 and G16; Fig. 5). Shear strain magnitudes between HOM and SUT models are very similar, with notable differences only apparent at G5 and G7 (higher in the SUT model, and closer to the experimental results), and G8 and G10 (lower in the SUT model, and further from the experimental results). Although G10 from the SUT model gives a result that is further from the experimental result absolutely, it has relatively lower values of γ_max_ than G9 and G11, thus giving a pattern of strain in this location that is more consistent with the experiment.

### Euclidean Distances

Euclidean Distance (ED) is a metric that allows comparisons to be made between datasets by showing how similar they are to one another (with shorter distances indicating higher similarity), and has been used effectively in studies of FE sensitivity [25], [41], [42]. Euclidean Distances between each model and the experiment, and between the two FE-models, are given in Table 3. For all considered metrics, the FE-models are more similar to each other than they are to the experimental data. In the case of principal and shear strains, this effect is considerable (an order of magnitude), though this is because the models are too stiff compared to the real bone. This effect is also observable in the strain orientations, although it is smaller. Interestingly, when considering ε_max_ magnitudes alone, the SUT model is closer to the experimental data, yet when considering ε_min_, the HOM model is closer.

**Table 3:** Euclidean distances between the two models, and between each model and the experimental data.

	Euclidean Distance		
	HOM - Exp.	SUT - Exp.	HOM - SUT
ε_max_	740.53	681.07	97.38
ε_min_	690.26	734.00	76.09
Total strain*	1012.35	1001.31	123.58
Strain Ratio	3.78	4.86	3.57
Orientation	69.96	74.03	47.51
Shear Strain	1278.62	1247.79	131.21

*Total strain = ED calculated from combined ε_max_ and ε_min_ values. Calculations exclude G1 & G6.

Strain ratio again is preferred as the metric for comparison, as the effects of strain magnitude are removed. Although the models are more similar to each other than to the experiment, the ED values suggest that the HOM model is almost as similar to the experiment (ED = 3.78) as it is to the other model (ED = 3.57). Additionally, the ED values show that the SUT model is less similar to the experimental results than the HOM model (ED = 4.86).

Further analysis of this phenomenon was conducted when it was noticed from the graph of strain ratio (Fig. 3) that most of the differences between models were observed in the zygomatic/facial region (G1–8, and particularly G4), whereas very few differences could be seen around the braincase (G9–16). Accordingly, the dataset was divided into “facial” and “cranial vault” subsets, and ED recalculated for each. The results presented in Tables 4 and 5 show that, in the cranial vault, the FE-models are indeed very similar to each other, and much more so than to the experimental results. The results for the facial region are broadly reflective of those for the whole model, although interestingly, and contra to the results of the whole dataset or cranial vault subset, show that strain ratio in the HOM model is more similar to the experiment than it is to the other model.

**Table 4:** Euclidean distances between the two models, and between each model and the experimental data in the facial/zygomatic region (G1–8).

	Facial/Zygomatic (G1–8)		
	Euclidean Distance		
	HOM - Exp.	SUT - Exp.	HOM - SUT
ε_max_	711.95	648.99	96.21
ε_min_	623.84	671.80	71.21
Total strain*	946.60	934.08	119.70
Strain Ratio	2.88	4.29	3.52
Orientation	40.50	55.50	35.64
Shear Strain	1189.79	1153.73	126.15

*Total strain = ED calculated from combined ε_max_ and ε_min_ values. Calculations exclude G1 & G6.

**Table 5:** Euclidean distances between the two models, and between each model and the experimental data in the cranial vault region (G9–16).

	Cranial Vault (G9–16)		
	Euclidean Distance		
	HOM - Exp.	SUT - Exp.	HOM - SUT
ε_max_	203.76	206.58	15.03
ε_min_	295.44	295.70	26.80
Total strain*	358.89	360.71	30.73
Strain Ratio	2.45	2.28	0.63
Orientation	57.05	48.99	31.42
Shear Strain	468.27	475.28	36.09

*Total strain = ED calculated from combined ε_max_ and ε_min_ values.

### Contour plots

**Figure 6:**
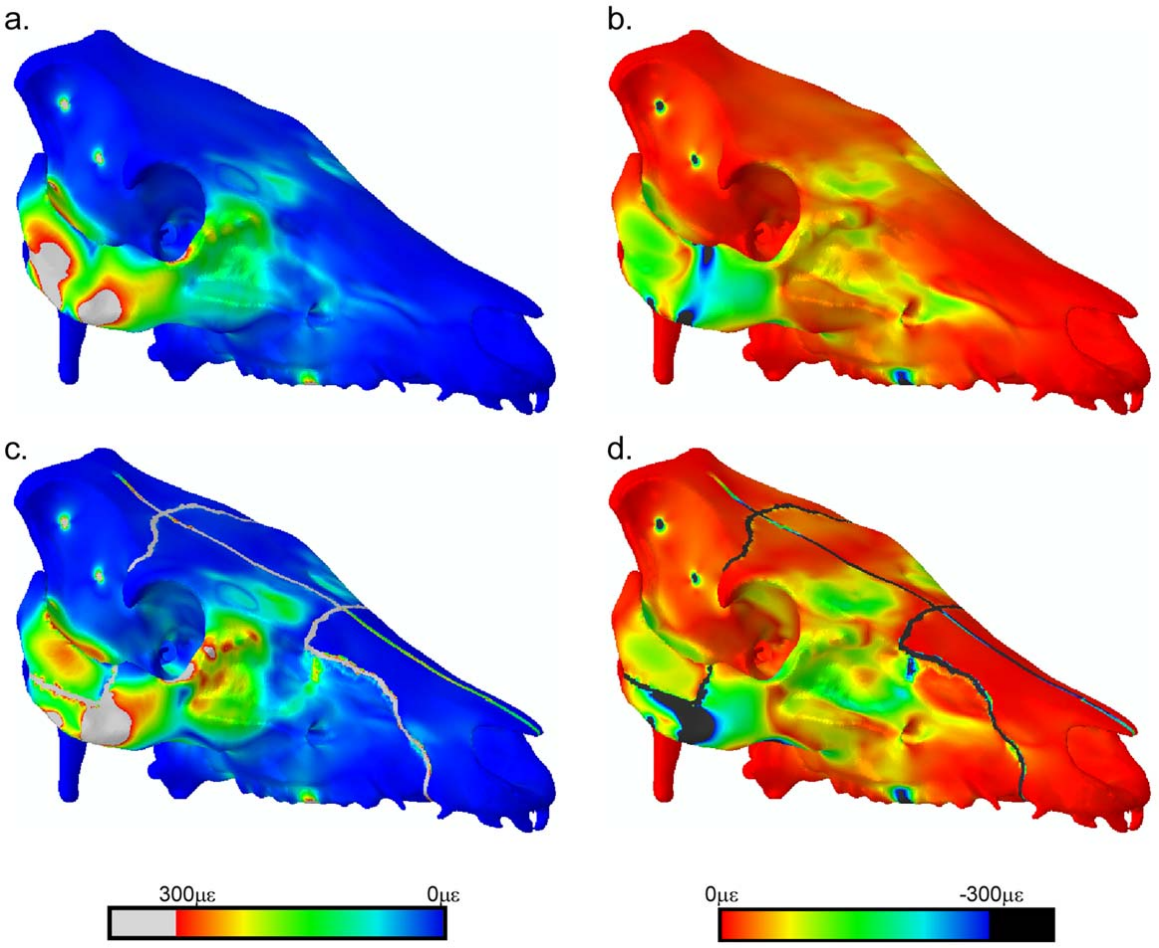
Principal strain contour plots capped at 0–300 με. Contour plots showing maximum (a, c) and minimum (b, d) principal strains, in models without (a, b) and with (c, d) sutures. Note that the maximum and minimum principal strains are capped between 0 με and +300 με or −300 με respectively.

**Figure 7:**
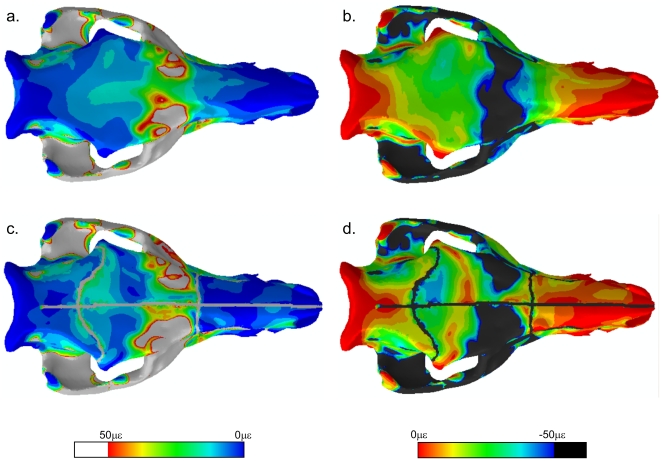
Principal strain contour plots capped at 0–50 με. Contour plots showing maximum (a, c) and minimum (b, d) principal strains, in models without (a, b) and with (c, d) sutures. Note that the maximum and minimum principal strains are capped between 0 με and +50 με or −50 με respectively.

Such quantitative analysis is restricted to the locations of the strain gauges. To see how sutures affect the whole model, principal strain contour plots were produced and compared qualitatively (Fig. 6). The sutures show up as regions of greatly increased local strain. There are some notable differences between the models in the facial region, particularly in the maxilla, where strains are higher in the SUT model, and in the zygomatic arch, where strains are increased anteriorly (and extending to the lacrimal bones) and decreased posteriorly in the SUT model. Slight increases in strain are also seen in the anterior frontal bone in the SUT model. Small differences in the cranial vault between the SUT and HOM models become apparent when the contours displaying maximum and minimum principal strain are re-scaled between 0 με and +50 or −50 με respectively (Fig. 7). The SUT model shows a band of decreased strain bilaterally across the frontals, but increased strain at the anterior and posterior frontal margins.

To summarise, G5 and G8 (zygomatic arch) and G7 (anterior frontal) give notably different results in the SUT and HOM models for ε_max_, ε_min_, and γ_max_ magnitudes, and strain ratio. Gauge 4 (dorsal maxilla) shows differences in principal strain magnitude and orientation, and strain ratio. Also affected are G10 [left frontal (γ_max_)], and G2/G11 [anterior nasal/cranial vault respectively (strain ratio)]. Gauges 3 (maxilla, dorsal to the loaded tooth), G4 (dorsal maxilla), G6 (posterior nasal), G9 (right frontal), and G12, G14, and G16 (cranial vault) show differences in strain orientation between the two models. The models are more similar to each other than they are to the experimental dataset, except in the case of strain ratio in the facial subset of gauges, where the HOM model is closer to the experiment than it is to the other model. Whether the HOM or SUT model is closer to the experimental data set depends both on which metric is being considered, and where the gauge is located.

## Discussion

### *Ex vivo* experimental results

The *ex vivo* experimental results are in keeping with other published works, showing that the strain environment can change substantially when moving from one bone to another across a suture [4]–[7], [13]–[17]. This is particularly true between the two bones of the zygomatic arch (G5, G8) and between the four frontal and parietal bones (G11–16). The right parietal (G12, G16; which experienced higher loading from the “working-side” temporalis) shows higher strain magnitudes, and bilaterally the parietals show a different strain orientation to the frontals (Figs. 2, 5). From this dataset then, one could reasonably conclude that the presence of sutures may be influencing *ex vivo* strains in the pig cranium by altering strain magnitudes and orientations.

### Sensitivity: comparisons between FE models with and without sutures

The graphs of principal and shear strain, and strain orientation, suggest that when sutures are included in the FE models few differences are observed in most locations when compared against the model without sutures. This seems to lead to the opposite conclusion of that stated in the previous paragraph: sutures exert little influence on the strain environment of the pig cranium. However, observation of the graph of strain ratio suggests that the situation is more complex than this, as some gauge locations, particularly G4, show distinct separation of the HOM and SUT models.

Analysis of the Euclidean Distances between the FE-models and the experiment for the metrics measured demonstrates that the two models are more similar to each other than either is to the experimental dataset. However, when strain magnitudes are removed by considering strain ratio, the HOM model is nearly as similar to the experiment as it is to the SUT model. This implies that the models are sensitive to the presence or absence of sutures; however this sensitivity is not consistent across the entire skull. Subdividing the results based on location shows that sutures exert a much greater influence in the “facial” region of the model, whereas differences in the cranial vault are limited. Different metrics also respond differently to the presence or absence of sutures. Some differences in the contour plots were apparent between the pig models, and these were similar to the results of Kupczik et al. [25] who also observed increased strain in the anterior zygomatic arch and lacrimals.

That the FE-model is sensitive to the presence of open cranial sutures agrees with other FE studies on lizards and alligators, which have indicated that sutures play an important role in FE analyses by changing the strain regimes between different bones [24], or by redistributing strains so that overall, the skull experiences a more even strain environment (which may not be apparent under *ex vivo* or static loading conditions [23]). Surprisingly, the findings of this study are contra to those of Wang et al. [26], who found only minor differences in strain pattern between their macaque models with and without sutures. Wang et al. [26] suggested that their result may be due to the fact that primates have relatively few craniofacial sutures, making up only a small percentage of the total skull volume, especially when compared to reptilian skulls. They stated that, due to the extensive *in vivo* research indicating that sutures play an important role in pigs “a comparison between primates and pigs…seems warranted” ([26]:1486). It is interesting to speculate on the reasons that the pig and macaque studies give opposite results. The fact that all of the differences between the HOM and SUT models are confined to the zygomatic and facial regions may provide a clue; the long snout of the pig is clearly a very different structure to the relatively flat face of primates, and the robust zygomatic arches of the pig experience incredibly high loads during feeding [4]. It may be that the large, prominent sutures of the pig snout are of different functional importance than those in the faces of primates, as pigs use their snouts in a range of foraging behaviours unrelated to mastication.

### Validation: comparisons between FE models and *ex vivo* experimental strains

The experimental results shown here, and those of previous works mentioned above, seem to indicate strongly that strains on adjacent bones can be very different, and by inference suggest that the relationship between sutures and strains is important. Furthermore, it has also been demonstrated that FE models can be sensitive to the presence of craniofacial sutures. However, the hypothesis that the inclusion of sutures in the model would improve the fit of the model to the experimental data was not borne out.

Models are, by definition, simplified approximations of reality. Finite Element Analysis is a mathematically robust technique with a long history of industrial engineering use [43]. Therefore, if FE models do not match with experimental data, then certain assumptions about model construction must be incorrect. The effect of sutures here was tested in isolation, and the inclusion of sutures in the model was often found to result in higher Euclidean Distances to the experiment than were seen with the HOM model. This means that including sutures did little to improve the fit of the models to the experimental data, and in many cases made the datasets more disparate. The lack of fit between the models and the experiment therefore cannot be attributed to the presence or absence of patent sutures alone. It is possible that the modelling technique used to incorporate the sutures was not realistic. Sutures were included as strips of more compliant 3D elements; an approach commonly used in other studies [19], [23]–[26], [41]. Importantly, this approach was recently demonstrated to be an effective method of modelling strains and displacements in the pig zygomatic arch [30], which contains both interdigitated and butt-ended suture morphologies that are loaded in both tension and compression. The transition between material properties from bone to suture when using this modelling technique is likely to be much more abrupt than the actual transition between bone and suture in the specimen, and the actual suture is much thinner than the band of elements. However, this technique has been shown to provide an accurate profile of the strain gradient between the two materials [30]. An overly thick band of sutural material could absorb a relatively high proportion of the model strain, and this could account for some of the discrepancy between the two datasets. However, the fact that the HOM model also fails to match the experiment suggests that it is unlikely that the suture modelling method is responsible for the strain magnitude inaccuracies of the FE-models.

An alternative explanation for the lack of fit between strain magnitudes in the FE-models and the experiment could be found in a consideration of the material properties applied to the model. A large part of this discrepancy has already been attributed to the incorrect practice of simplifying bone to a material with isotropic, homogeneous properties, although bone is in actuality a far more complicated structure than this [31]. Cranial bone in particular is known to vary in stiffness among and within bones, and depending on the axis in which the loading is applied [34], [44], [45]. A number of studies have indicated that biological FE models are highly sensitive to choices of bone material properties, often more so than any other input parameter [24], [27], [31], [46]. Additionally, failure to consider variation in the material properties of the sutures themselves may account for some of the discrepancy, as the levels of fusion and anisotropy encountered in each individual suture was not known. Even though most gauge locations replicate strain ratio and orientation reasonably well, in some locations (such as G4), both FE-models can be far from the experimental results. It is therefore possible that the true effects of sutures on model validity in these locations are being masked by incorrect assumptions in other modelling parameters.

Without detailed material properties data from pig skulls, it is impossible to say at this stage whether the experimental pattern of strain changes across sutures is due to the sutures themselves, or different material properties being present in the two bones on either side of the suture. Because the pig specimen used was sub-adult and had not yet reached skeletal maturity, growth was therefore still occurring at the sites of the craniofacial sutures. Regions of less ossified bone were therefore probably present in the cranium [47], and as the centres of growth, it is likely that these would occur near the sutures. If the observed effect of changing strains in adjacent bones is actually caused by local differences in material properties within and among bones, rather than being an effect of the suture itself, this might explain the difference between the experimental and modelling results, although this has not been tested here. Finite Element Analysis alone cannot answer this question, and more experimental data on strain and material properties throughout ontogeny would be most useful.

In conclusion, it can be demonstrated that the models presented were sensitive to the presence of cranial sutures, and that different regions of the skull display differences in sensitivity to the presence of sutures, with the zygomatic and facial regions more sensitive than the cranial vault. However, introducing sutures to the model often resulted in a model that was less accurate than one which excluded them. Even after extensive validation work, neither the HOM nor SUT model can be considered valid in terms of strain magnitude, although experimental strain ratios and orientations, and therefore overall strain regime, can be replicated by the FEA in most locations. Neither FE-model could therefore be used to draw conclusions on pig skull function. This should serve to caution workers who are unable to validate their models, particularly palaeontologists, for whom vast amounts of input information are missing.
